# Supplementary material for: Hot spot prediction in protein-protein interactions by an ensemble system
Source: BMC Syst Biol. 2018 Dec 31;12(Suppl 9):132. doi: 10.1186/s12918-018-0665-8 (PMC6311905; doi:10.1186/s12918-018-0665-8)
Supplement: Supplementary file 2 — All relevant MATLAB code. A simple Matlab implement of our predictor. (ZIP 19,742 kb) [file 12918_2018_665_MOESM2_ESM.zip › Additional file 2/Read ASA/ASA website/ASEdb/1DVFD.htm]

 NetSurfP - Protein Surface Accessibility and Secondary Structure Predictions

  

|  |  |  |
| --- | --- | --- |
|  |  | NetSurfP - Protein Surface Accessibility and Secondary Structure Predictions  Technical University of Denmark |

  


---

```
# For publication of results, please cite:
# A generic method for assignment of reliability scores applied to solvent accessibility predictions.
# Bent Petersen, Thomas Nordahl Petersen, Pernille Andersen, Morten Nielsen and Claus Lundegaard
# BMC Structural Biology 2009, 9:51 doi:10.1186/1472-6807-9-51
#
# Column 1: Class assignment - B for buried or E for Exposed - Threshold: 25% exposure, but not based on RSA
# Column 2: Amino acid
# Column 3: Sequence name
# Column 4: Amino acid number
# Column 5: Relative Surface Accessibility - RSA
# Column 6: Absolute Surface Accessibility
# Column 7: Z-fit score for RSA prediction
# Column 8: Probability for Alpha-Helix
# Column 9: Probability for Beta-strand
# Column 10: Probability for Coil
E Q  1DVFD                  1    0.732 130.664   1.183   0.003   0.003   0.994
B V  1DVFD                  2    0.204  31.416  -0.242   0.004   0.616   0.381
E Q  1DVFD                  3    0.398  71.119   1.158   0.002   0.816   0.182
B L  1DVFD                  4    0.156  28.600   0.665   0.018   0.846   0.136
E Q  1DVFD                  5    0.357  63.778   0.629   0.021   0.756   0.223
B Q  1DVFD                  6    0.233  41.632   0.769   0.022   0.552   0.426
E S  1DVFD                  7    0.376  44.032  -0.466   0.020   0.205   0.775
E G  1DVFD                  8    0.427  33.581  -1.775   0.018   0.088   0.893
E T  1DVFD                  9    0.506  70.182  -2.183   0.018   0.047   0.935
E E  1DVFD                 10    0.487  85.009  -1.677   0.019   0.141   0.840
E L  1DVFD                 11    0.353  64.671  -0.688   0.021   0.279   0.699
E V  1DVFD                 12    0.312  48.016   0.471   0.022   0.359   0.619
E K  1DVFD                 13    0.402  82.691   1.002   0.020   0.205   0.775
E S  1DVFD                 14    0.459  53.760  -0.698   0.018   0.047   0.935
E G  1DVFD                 15    0.595  46.811  -2.146   0.018   0.047   0.935
E A  1DVFD                 16    0.425  46.846  -0.906   0.004   0.085   0.910
E S  1DVFD                 17    0.404  47.372  -0.713   0.005   0.336   0.660
B V  1DVFD                 18    0.155  23.793   0.736   0.003   0.718   0.279
E K  1DVFD                 19    0.393  80.840   0.694   0.001   0.900   0.099
B L  1DVFD                 20    0.081  14.740   0.462   0.001   0.900   0.099
B S  1DVFD                 21    0.145  17.041   0.713   0.001   0.900   0.099
B C  1DVFD                 22    0.043   5.967   0.157   0.001   0.900   0.099
B T  1DVFD                 23    0.279  38.697   0.527   0.001   0.900   0.099
B A  1DVFD                 24    0.107  11.747  -0.213   0.003   0.718   0.279
B S  1DVFD                 25    0.180  21.108  -0.685   0.004   0.420   0.576
B G  1DVFD                 26    0.246  19.376  -1.563   0.004   0.138   0.858
B F  1DVFD                 27    0.263  52.744  -0.955   0.021   0.279   0.699
B N  1DVFD                 28    0.169  24.712  -1.106   0.021   0.451   0.528
B I  1DVFD                 29    0.108  19.999  -1.127   0.021   0.451   0.528
B K  1DVFD                 30    0.212  43.547  -0.940   0.021   0.279   0.699
B D  1DVFD                 31    0.288  41.573  -1.079   0.019   0.141   0.840
B T  1DVFD                 32    0.107  14.785  -0.126   0.020   0.205   0.775
B H  1DVFD                 33    0.082  14.989   0.134   0.022   0.359   0.619
B M  1DVFD                 34    0.026   5.143   0.437   0.021   0.756   0.223
B N  1DVFD                 35    0.048   6.998  -0.181   0.018   0.846   0.136
B W  1DVFD                 36    0.032   7.744   0.467   0.018   0.846   0.136
B V  1DVFD                 37    0.037   5.702   0.312   0.018   0.846   0.136
B K  1DVFD                 38    0.126  25.836   0.462   0.021   0.756   0.223
B Q  1DVFD                 39    0.093  16.628   0.322   0.022   0.359   0.619
B R  1DVFD                 40    0.217  49.670   0.649   0.005   0.045   0.951
E P  1DVFD                 41    0.357  50.715   0.624   0.005   0.015   0.979
E E  1DVFD                 42    0.543  94.810  -1.636   0.018   0.019   0.964
E Q  1DVFD                 43    0.424  75.691   0.514   0.018   0.019   0.964
E G  1DVFD                 44    0.469  36.918  -0.434   0.018   0.088   0.893
B L  1DVFD                 45    0.172  31.530   0.966   0.005   0.336   0.660
B E  1DVFD                 46    0.112  19.654  -0.427   0.003   0.718   0.279
B W  1DVFD                 47    0.049  11.760   0.283   0.001   0.900   0.099
B I  1DVFD                 48    0.024   4.514   0.948   0.001   0.959   0.040
B G  1DVFD                 49    0.017   1.354  -0.008   0.001   0.959   0.040
B R  1DVFD                 50    0.084  19.350  -0.539   0.001   0.959   0.040
B I  1DVFD                 51    0.023   4.255   0.903   0.001   0.959   0.040
B D  1DVFD                 52    0.154  22.191   0.717   0.003   0.718   0.279
B P  1DVFD                 53    0.297  42.187  -0.933   0.005   0.262   0.733
E A  1DVFD                 54    0.591  65.084  -2.276   0.005   0.015   0.979
E N  1DVFD                 55    0.602  88.162  -2.785   0.016   0.005   0.979
E G  1DVFD                 56    0.518  40.790  -2.097   0.018   0.019   0.964
E N  1DVFD                 57    0.472  69.115  -0.555   0.018   0.088   0.893
B I  1DVFD                 58    0.258  47.767   0.032   0.020   0.205   0.775
E Q  1DVFD                 59    0.407  72.672   0.244   0.021   0.279   0.699
B Y  1DVFD                 60    0.211  44.984   0.560   0.020   0.205   0.775
B D  1DVFD                 61    0.200  28.878  -0.498   0.052   0.084   0.864
E P  1DVFD                 62    0.638  90.504  -0.545   0.053   0.043   0.903
E K  1DVFD                 63    0.421  86.517   0.177   0.052   0.084   0.864
B F  1DVFD                 64    0.084  16.839  -0.108   0.019   0.141   0.840
E R  1DVFD                 65    0.400  91.508   0.923   0.019   0.141   0.840
B G  1DVFD                 66    0.254  19.958  -1.627   0.005   0.262   0.733
B K  1DVFD                 67    0.235  48.257   0.830   0.003   0.718   0.279
B A  1DVFD                 68    0.053   5.841  -0.246   0.001   0.900   0.099
B T  1DVFD                 69    0.128  17.809   0.897   0.001   0.959   0.040
B I  1DVFD                 70    0.042   7.696   0.801   0.001   0.959   0.040
B T  1DVFD                 71    0.119  16.436   0.327   0.002   0.816   0.182
B A  1DVFD                 72    0.249  27.484   0.595   0.004   0.514   0.481
B D  1DVFD                 73    0.271  39.022  -0.149   0.004   0.420   0.576
E T  1DVFD                 74    0.461  63.927   0.039   0.018   0.088   0.893
B S  1DVFD                 75    0.246  28.866  -1.899   0.018   0.047   0.935
E S  1DVFD                 76    0.533  62.503   0.951   0.056   0.142   0.802
E N  1DVFD                 77    0.265  38.840  -0.203   0.021   0.279   0.699
B T  1DVFD                 78    0.199  27.615   0.945   0.021   0.756   0.223
B A  1DVFD                 79    0.073   8.100  -0.714   0.011   0.918   0.071
B Y  1DVFD                 80    0.171  36.564   0.448   0.011   0.918   0.071
B L  1DVFD                 81    0.049   8.917   0.644   0.011   0.918   0.071
B Q  1DVFD                 82    0.142  25.290  -0.328   0.018   0.846   0.136
B L  1DVFD                 83    0.076  13.916  -0.444   0.022   0.552   0.426
E S  1DVFD                 84    0.345  40.387  -0.162   0.021   0.279   0.699
E S  1DVFD                 85    0.361  42.309  -0.862   0.019   0.141   0.840
B L  1DVFD                 86    0.094  17.230   0.134   0.018   0.088   0.893
E T  1DVFD                 87    0.464  64.357   1.050   0.018   0.047   0.935
E S  1DVFD                 88    0.359  42.075  -0.531   0.052   0.084   0.864
E E  1DVFD                 89    0.420  73.357  -0.564   0.053   0.043   0.903
B D  1DVFD                 90    0.163  23.431  -0.955   0.052   0.084   0.864
B T  1DVFD                 91    0.108  14.938   0.219   0.069   0.386   0.545
B A  1DVFD                 92    0.037   4.077  -0.204   0.021   0.756   0.223
B V  1DVFD                 93    0.061   9.330   1.070   0.018   0.846   0.136
B Y  1DVFD                 94    0.032   6.796   0.946   0.011   0.918   0.071
B Y  1DVFD                 95    0.047  10.087   0.957   0.018   0.846   0.136
B C  1DVFD                 96    0.014   2.022   1.621   0.021   0.756   0.223
B A  1DVFD                 97    0.028   3.119   0.183   0.022   0.552   0.426
B T  1DVFD                 98    0.110  15.243   0.349   0.021   0.279   0.699
B K  1DVFD                 99    0.254  52.186  -0.525   0.018   0.088   0.893
B V  1DVFD                100    0.291  44.650  -1.023   0.018   0.047   0.935
E I  1DVFD                101    0.398  73.574  -2.275   0.018   0.047   0.935
E Y  1DVFD                102    0.317  67.636  -2.162   0.018   0.088   0.893
B Y  1DVFD                103    0.289  61.823  -2.968   0.018   0.088   0.893
B Q  1DVFD                104    0.300  53.598  -2.220   0.018   0.047   0.935
B G  1DVFD                105    0.291  22.941  -2.224   0.018   0.047   0.935
B R  1DVFD                106    0.305  69.891  -1.773   0.005   0.015   0.979
B G  1DVFD                107    0.245  19.266  -1.050   0.018   0.047   0.935
B A  1DVFD                108    0.086   9.455  -0.793   0.018   0.088   0.893
B M  1DVFD                109    0.043   8.564  -0.321   0.019   0.141   0.840
B D  1DVFD                110    0.069   9.986  -0.680   0.019   0.141   0.840
B Y  1DVFD                111    0.061  13.100  -0.786   0.019   0.141   0.840
B W  1DVFD                112    0.041   9.861  -0.298   0.019   0.141   0.840
B G  1DVFD                113    0.041   3.250  -2.107   0.019   0.141   0.840
B Q  1DVFD                114    0.149  26.522   0.145   0.004   0.138   0.858
B G  1DVFD                115    0.124   9.751  -1.395   0.005   0.262   0.733
B T  1DVFD                116    0.104  14.397   0.063   0.004   0.514   0.481
E T  1DVFD                117    0.282  39.169   0.279   0.002   0.816   0.182
B L  1DVFD                118    0.068  12.432  -0.033   0.001   0.900   0.099
B T  1DVFD                119    0.215  29.751   0.023   0.002   0.816   0.182
B V  1DVFD                120    0.169  25.945  -0.197   0.003   0.718   0.279
E S  1DVFD                121    0.600  70.273  -0.939   0.005   0.015   0.979

Explain the output.  Go back.
```

---
